# A Wireless Optogenetic Headstage with Multichannel Electrophysiological Recording Capability

**DOI:** 10.3390/s150922776

**Published:** 2015-09-09

**Authors:** Gabriel Gagnon-Turcotte, Alireza Avakh Kisomi, Reza Ameli, Charles-Olivier Dufresne Camaro, Yoan LeChasseur, Jean-Luc Néron, Paul Brule Bareil, Paul Fortier, Cyril Bories, Yves de Koninck, Benoit Gosselin

**Affiliations:** 1 Department of Electrical and Computer Engineering, Université Laval, Quebec, QC G1V 0A6, Canada; E-Mails: gabriel.gagnon-turcotte.1@ulaval.ca (G.G.-T.); avakh.alireza@gmail.com (A.A.K.); reza.ameli@ymail.com (R.A.); charles-olivier.dufresne-camaro.1@ulaval.ca (C.-O.D.C.); paul.fortier@GEL.ULAVAL.CA (P.F.); 2 Doric Lenses Inc., Quebec, QC G1P 4N7, Canada; E-Mails: yoan@doriclenses.com (Y.L.); luc@doriclenses.com (J.-L.N.); paul@doriclenses.com (P.B.B.); 3 Institut Universitaire en Santé Mentale de Québec, Quebec, QC G1J 2G3, Canada; E-Mails: cyril.bories@gmail.com (C.B.); yves.dekoninck@neuro.ulaval.ca (Y.K.); 4 Centre d’Optique, Photonique et Laser (COPL), Université Laval, Quebec, QC G1V 0A6, Canada; 5 Department of Psychiatry & Neuroscience, Université Laval, Quebec, QC G1V 0A6, Canada

**Keywords:** neural headstage, optogenetics, neural recording, optical stimulation, wireless sensor, brain-computer interfaces, low-power biotelemetry, electrophysiology

## Abstract

We present a small and lightweight fully wireless optogenetic headstage capable of optical neural stimulation and electrophysiological recording. The headstage is suitable for conducting experiments with small transgenic rodents, and features two implantable fiber-coupled light-emitting diode (LED) and two electrophysiological recording channels. This system is powered by a small lithium-ion battery and is entirely built using low-cost commercial off-the-shelf components for better flexibility, reduced development time and lower cost. Light stimulation uses customizable stimulation patterns of varying frequency and duty cycle. The optical power that is sourced from the LED is delivered to target light-sensitive neurons using implantable optical fibers, which provide a measured optical power density of 70 mW/mm^2^ at the tip. The headstage is using a novel foldable rigid-flex printed circuit board design, which results into a lightweight and compact device. Recording experiments performed in the cerebral cortex of transgenic ChR2 mice under anesthetized conditions show that the proposed headstage can trigger neuronal activity using optical stimulation, while recording microvolt amplitude electrophysiological signals.

## 1. Introduction

Optogenetics is a neural stimulation technique that allows activation or deactivation and sensing [1] of specific light-sensitive neurons with high temporal and spatial accuracy [2,3]. This technique is based on introducing light-responsive proteins into neurons through genetic engineering. When the proteins are light-gated ion channels, the approach allows activation of ionic conductance across the cell membrane with light, which provides researchers with a strong tool of crucial importance for brain-computer interface development. Applications range from selective control of specific brain regions for identifying their function, to neuroprosthetics, and even treatment of diseases related to dysfunctions of certain parts of the nervous system [2]. Besides, the necessity of having a tightly coupled electrophysiological recording readout to monitor the evoked brain activity at the single-neuron for tracking the effect of optical neural activation has been highlighted [4].

Recently, optogenetics has been used to carry various experiments in freely-behaving rodents, especially mice [5,6,7,8,9,10], which serve as disease models. As a result, many research groups have worked on optogenetics-related hardware to address the growing need in this area [5,6,7,8,9,10]. Since the nature of these experiments requires flexible tools, which can evolve over time, the need for new optogenetic systems is always present. In particular, optical stimulation systems that can simultaneously record brain activity for allowing real time readout of light effect on brain activity, especially in freely moving animals, are highly sought [11]. At the time of this writing, only a few commercial and research headstage systems are available [5,6,7,8,9,10]. These systems present different characteristics, such as electrophysiological recording and optical stimulation [5,6,7,8], high-power lasers or diodes for optical stimulation [5,6,7,8], and wireless or wired transmitters [5]. However, commercial wireless systems, such as those proposed in [6,7], either provides optical stimulation from a maximum of two channels or electrophysiological recording from several channels (from up to 126 channels), but not both functions. Similarly, existing wirelessly powered implantable optoelectronic devices, such as the one proposed in [8], can offer cellular-scale optical stimulation using inorganic light-emitting diodes (µ-ILEDs) but do not provide any electrophysiological recording means. The system presented in [9] provides 32 electrical stimulation channels but does not include any electrophysiological recording circuitry. Although many of these tools and settings have interesting features, to our knowledge, none of them benefit from combined implantable fiber-coupled LED and multiple electrophysiological recording channels while possessing wireless transmission. The ones featuring optical stimulation LED or lasers are either tethered to a power source via cables [6,7], and/or are missing electrophysiological recording capability [8], while the ones capable of high-quality signal recording lack light stimulation circuitry [12,13].

Designing a wireless optogenetic headstage is a challenging task as there are many design constraints and considerations that have to be taken into account. Two of the most challenging constraints are: the limited energy source and the need for a small form factor. More specifically, limited power sources such as batteries and wireless power delivery links [5,14,15] create serious limitations on the amount of optical power that can be delivered to the target light-sensitive neurons. In addition, the size requirement of the optogenetic headstage, especially when designed for mice, results in a more compact size and smaller power sources. Other challenges to designing a neural headstage, with or without optical stimulation, involve electromagnetic compatibility (EMC) and electromagnetic interference (EMI) issues. A wireless optogenetic headstage must include ultra-low noise analog circuitry [16,17,18], high-current optical stimulation circuitry [5,6,7], digital and mixed-signal processors [5,19] and radio frequency (RF) transmission circuitry. Combining all these building blocks together inside a small package can cause EMC/EMI issues that can significantly degrade the performance of the headstage. The high switching currents in the optical stimulation circuits, as well as charge injection and clock feedthrough from the digital circuits, can cause high fluctuations in the power supply rails, and induce noise in the sensitive analog circuitry.

In this paper, we present an optogenetic headstage incorporating two high-power LED and two electrophysiological readout channels. The headstage is built entirely using low-cost commercial off-the-shelf (COTS) components for better flexibility, lower cost and reduced development time. It can drive each LED with up to 150 mA resulting in 8 mW output optical power from a 200-μm core implanted optical fiber (250 mW/mm^2^) to deliver light to the target brain regions. Each of the two readout channels, also referred to as an analog front end (AFE), amplify and filter the acquired neural signal. A commercial radio transceiver operating at a center frequency of 2.4 GHz is used to transmit the acquired and digitized neural signals back to a base station computer where more processing is performed in the captured signals. The system is powered using a small lithium-ion battery and can continuously operate for up to 3 h. A MSP430 microcontroller from Texas Instruments equipped with a real-time operating system (RTOS) is in charge of system control, digitizing the AFE outputs and data transmission. To the best of our knowledge, this is the first reported fully wireless headstage to offer simultaneous multichannel optical stimulation along with multichannel brain signals recording capability. In Section 2 of this paper, an overview of the proposed optogenetic headstage is presented and all building blocks and rationale behind their design are described in detail. In Section 3, we present *in vitro* and *in vivo* measurements obtained with the fabricated headstage prototype. *In vivo* trials were performed with a transgenic mouse expressing Channelrhodopsin-2. Section 4 includes the conclusion and future work.

## 2. System Overview and Design

**Figure 1:**
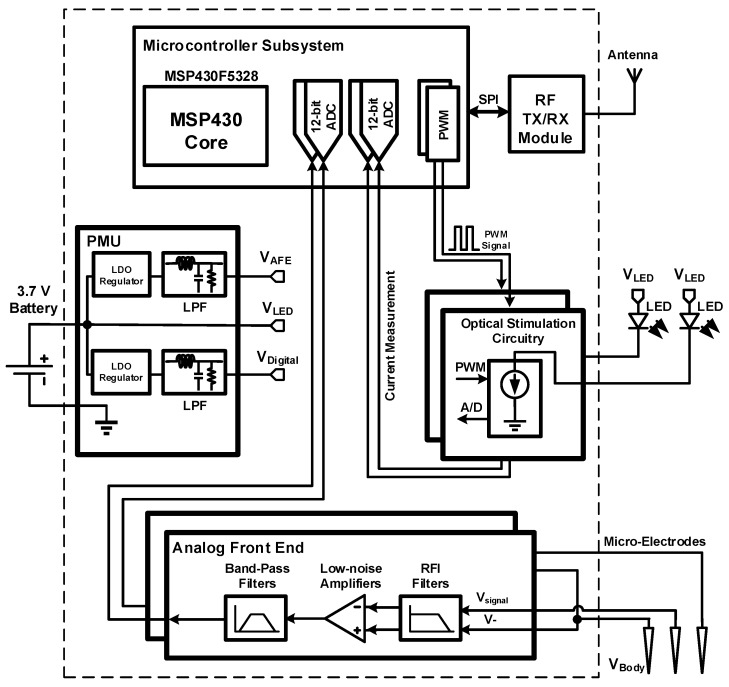
Block diagram of the proposed headstage system.

The proposed wireless optogenetic system, the block diagram of which is shown in Figure 1, consists of two interconnected parts. The first part is the headstage itself, which contains the electronics for powering the LED, for recording and amplifying neural signals and for wirelessly transferring neural data to a base station. The second part consists of an implantable module, which holds the LED, the optical fibers and two microelectrodes. The headstage system includes five main building blocks: (1) the analog front-end; (2) the optical stimulation circuitry; (3) the power management unit (PMU); (4) the wireless transceiver; and (5) the mixed-signal controller. The AFE amplifies the low-amplitude action potentials (AP) whose bandwidth lies between 300 Hz and 7 kHz [16]. Very high common-mode rejection ratio (CMRR) and power supply rejection ratio (PSRR) are necessary for a smooth and reliable neural signal acquisition where the expected amplitudes of the signals are very low [16,17,18,19]. The AFE achieves its high PSRR partly by incorporating electronic components with high PSRR specifications and partly by having a separate power supply filter network, which removes fast power supply fluctuations.

The role of optical stimulation circuitry is to apply a determined voltage pattern across the stimulation LED pins. This voltage pattern, which directly translates to the LED current, needs to be precise in order to ensure that the optical power delivered to the target neurons is accurate. The presented optogenetic headstage can deliver a current of 150 mA to the stimulation LED [20] using precise closed-loop current sources. The PMU is responsible for supplying a 3.3-V supply voltage for the control unit and the radio transceiver. The PMU incorporates a power supply filter, which removes high-frequency fluctuations in the regulated supply voltages. Otherwise, these high-frequency fluctuations would be observable in the supply voltage from the battery when the optical stimulation LED are active. A digital radio transceiver working at 2.4 GHz is used to transmit the acquired data to a base station, and to receive the stimulation parameters. The control unit is based on a low-power microcontroller (MCU) from the MSP430 family of Texas Instruments. This microcontroller generates the optical stimulation patterns, digitizes the output signals of the AFE, transmits data using the radio transceiver, and, in general, controls the functionality of the whole headstage system.

The headstage is connected with biological tissues through an implantable module, which is a mechanical part that is mounted on the rodent’s head by surgery holding in place with dental cement; this component includes, the optical fibers and the microelectrodes both connected to the headstage through a Molex board-to-board connector. In the following subsections, we will describe each building block of the headstage in detail, including the implantable module.

### 2.1. Implantable Module (LED and Microelectrodes)

The headstage is connected to the implantable module via a low-profile board-to-board Molex connector, enabling easy disconnection of the system between experiments and to recharge the battery. The fiber-coupled LEDs (Doric Lenses, Québec, QC, Canada) are connected to a printed circuit board inside the implantable module. Each LED is coupled with a 200-µm core optical fiber, and pre-packaged with two microelectrodes inside a thin polyimide tubing of 400 µm outer diameter to minimize brain damage upon implantation. Tungsten microelectrodes from MicroProbes (Gaithersburg, MD, USA) having a shank diameter of 75 µm, and an impedance of 1 MΩ are employed for single-cell recording. Once the optrode (combined optical fiber and microelectrodes) is precisely placed into the brain, the implantable module must be cemented using dental cement on the animal skull to avoid optrode tethering and displacement, and to hold firmly the headstage in place. When used within *in vivo* experiments, the implantable module can be cemented to the skull of the animal to be held in place.

### 2.2. Analog Front End

**Figure 2:**
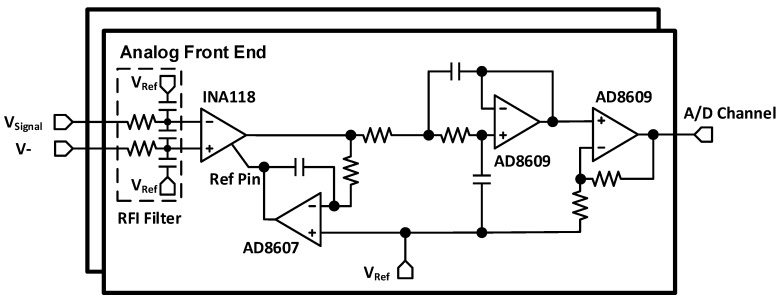
Schematic of the two-channel analog front-end.

The AFE is responsible for amplifying and conditioning the neural signal measured by the microelectrodes. Neural signals can be acquired using different methods [16], and, depending on the method, the amplitude of the acquired signals and the complexity and duration of the *in vivo* experimental protocol are different [16]. In this work, we aim to capture extracellular action potentials [5,16] with microelectrodes. In this scenario, it is expected that the amplitudes of the action potentials are between 50 µV and 150 µV [5,16,17]. As a result, an ultra-low-noise amplifier design with high CMRR and PSRR is necessary. Our design, based on low-cost commercial components, uses a high-performance INA118 instrumentation amplifier alongside a very low-noise LM4140 voltage reference, both from Texas Instruments. In addition, the AFE contains a 2nd order low-pass Sallen-key filter, a non-inverting amplifier, and a passive power supply filter network for removing high-frequency fluctuations, plus one low-dropout (LDO) voltage regulator (TLV70230, Texas Instruments, Dallas, TX, USA). Figure 2 shows the AFE circuitry. An ultra-low-noise 1.25-V voltage reference (LM4140) generates the mid-supply common-mode voltage for the instrumentation amplifier and other op-amps. Using such a low-noise and high-PSRR voltage reference is critical since this voltage directly appears at the inputs of the AFE, rendering it very sensitive to any noise or fluctuations that could be superimposed on the neural signal [21].

An overall gain of 3000 V/V and a −3 dB bandwidth between 300 Hz and 7 KHz, for full-bandwidth neural signal acquisition, is chosen for the AFE. It should be noted that the gain of 3000 V/V is suitable for amplifying input signals of amplitude of up to approximately 800 µV before saturation at the input of the analog-to-digital converter (ADC), because of an internal 2.5-V reference voltage inside the MSP430).

### 2.3. Optical Stimulation Circuitry

**Figure 3:**
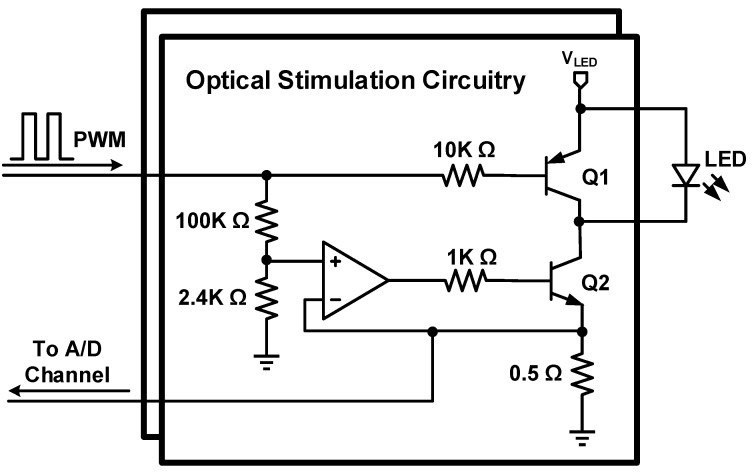
LED driver circuitry.

The stimulation LED are driven by the optical stimulation circuitry. This circuit uses a precise current source design based on a closed-loop op-amp to ensure the right amount of current is passed through the LED. The voltage across the 0.5-Ω resistor is fed to one ADC of the microcontroller transmitted to the base station to precisely monitor the optical stimulation timings, and to give a real-time feedback of the amount of current flowing into each LED during the experiment. Transistor Q1 (Figure 3) is used to make sure that the LED is turned off completely when there is no activation signal at the base of Q1 *i.e.*, the pulse width modulation (PWM) signal is on its low level. Figure 3 depicts the LED driver; since the LED are directly connected to the battery to benefit from a high forward voltage, power supply filter networks are implemented for every building block in the headstage in order to filter out any transient currents occurring upon activation of optical stimulation LEDs that would otherwise disturb the voltage supply of other circuits.

### 2.4. Power Management Unit

**Figure 4:**
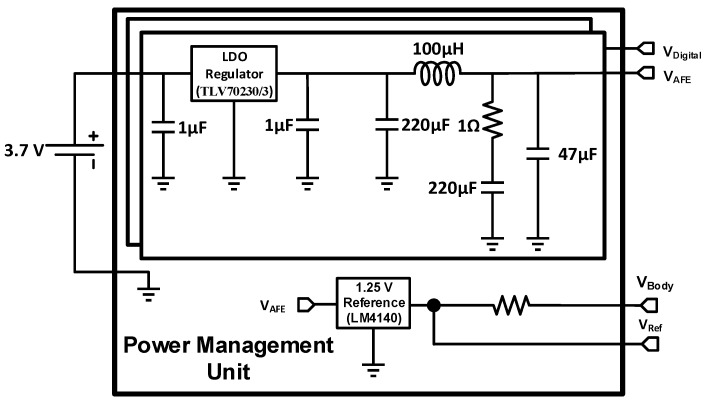
PMU circuitry.

Figure 4 shows the PMU circuitry. The whole headstage is powered by a 3.7-V, 100-mAh lithium-ion battery, so the PMU must provide a fixed 3.3-V supply voltage for the control unit and radio transmission. To address this requirement, a low-drop voltage regulator (TLV70233 from Texas Instruments) is used, which has a 51-dB PSRR to decrease the effects of high frequency fluctuations produced by optical stimulation circuitry. In addition, a passive power supply filter network is designed to be located after the regulation circuitry to remove the optical fluctuation effects. This passive network has a low-pass behavior and compensates the low PSRR of the LDO voltage regulator at higher frequencies.

In addition, as mentioned above, another low-drop regulator (TLV70230 from Texas Instruments) provides the required supply voltage in the AFE circuit, and an ultra-low-noise 1.25 V voltage regulator (LM4140) is embedded to produce the common-mode voltage. This common voltage has been chosen to be half of the internal ADC reference of the MSP430.

### 2.5. Wireless Transceiver

Wireless communications are insured by the ultra-low-power nRF24L01+ chip from Nordic Semiconductor. The chip integrates a complete 2.4-GHz Gaussian frequency-shift keying (GFSK) RF transceiver and synthesizer capable of transmitting at a maximum rate of 2 Mbps. Tests carried with this wireless transmitter have shown a measured maximum effective transmission rate of 0.7 Mbps. Such a transmission rate enables one to transmit as much as two channels of neural signals, each sampled at 20 kHz, and quantified on a maximum of 12 bits, corresponding to a maximum bit rate of 0.64 Mbps, which is below the aforementioned effective transmission rate. Control and configuration data of the transceiver are passed by the microcontroller via an 8-MHz Serial Peripheral Interface (SPI) bus. When transmitting at 0 dBm, the chip consumes as low as 11.3 mA, while consuming 13.5 mA in receiving mode at 2 Mbps. A quad-flat no-leads (QFN) 4 × 4-mm package allows this transceiver to be fully suitable for a compact PCB design.

### 2.6. Mixed-Signal Control System

**Figure 5:**
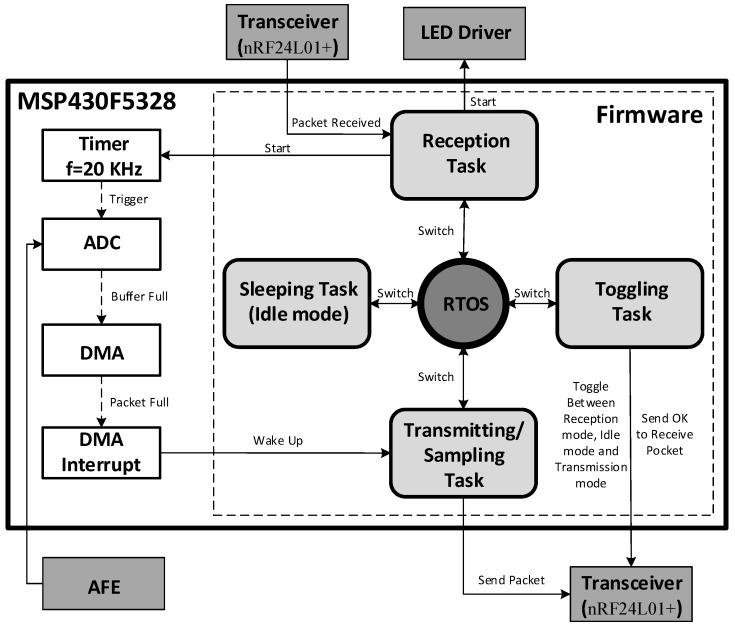
Functional diagram of the control firmware.

Digitization, control of RF communications, and generation of LED stimulation patterns are carried by a MSP430F5328 microcontroller from Texas Instrument [22]. To be able to use the microcontroller resources at its maximum of efficiency and the lowest power, a real-time operating system (RTOS) has been integrated into the control firmware. We selected the FunkOS [23] developed by Funkenstein Software Consulting since it supports all the needed functionalities for this application, while being fast and energy-efficient. RTOS, like the TinyOS [24], have already been used in previous neural recording systems. Unlike TinyOS, which uses the nesC programing language, FunkOS uses C language, which is a widespread programming language that is compatible with the MSP430 driver libraries. It is also fully compatible with the native interruptions of the microcontroller. The context switching latency was measured to be around 35 µs with an operating frequency of 8 MHz. In order to compare FunkOS with other RTOS, measurements were performed on the SYS/BIOS, the FreeRTOS and the BRTOS, which resulted in 94 µs, 137 µs and 42 µs of context switching latency, respectively. As previously mentioned, all the various tasks of the RTOS can be jointly used with the native interruptions of the system, allowing quick responses to any event, which is crucial to allow a sampling frequency of 20 kHz in the headstage, using the ADC and the DMA module available in the MSP430. So, after filling the buffer of the ADC, the DMA is triggered to transfer data from the ADC buffer directly to a packetizing module, before being passed to the transceiver and transmitted to the base station. When a packet is ready, the DMA interruption triggers the data transmission task via an RTOS semaphore. Thus, the MCU can execute other tasks or get into Idle mode between each packet by executing a Sleeping task (Figure 5).

The control firmware, the functional diagram of which is shown in Figure 5, consists in four main tasks. The task having the highest priority is dedicated to receiving configuration packets from the base station. Since the transceiver consumes more power in receiving mode (13.5 mA at 2 Mbps) than in transmitting mode (11.3 mA at 0 dBm), a task has been dedicated to toggle the transceiver from the receiving mode to the idle mode whenever possible. This task also transmits a synchronization packet and sets the transceiver in receiving mode for a short period of time. If no configuration packet has been received, the transceiver is switched into idle mode before repeating the cycle. Another task is dedicated to transmitting the captured neural data to the transceiver when the DMA finishes filling the buffer packet. A sleeping task is dedicated to put the microcontroller in idle mode for saving power when no other task has to be executed. Such a sleeping task saves power by turning off the MCU when it is not in use. The MCU is brought back in the active mode by using interrupts. With a packet size of 32 bytes, the Sampling/Transmitting task is triggered by a DMA interruption every 800 µs. Note that changing the task context requires a 35-µs delay. A 70-µs delay is required to switch from the Sleeping task to the Sampling/Transmitting task and vice versa. Additionally, transferring a packet to the transceiver requires around 42 µs using a SPI link at 8 MHz. As a result, the MCU can ideally be in the sleeping task for 688 µs between DMA interrupts (800-µs periods), which corresponds to 86% of the CPU time, the context changes being automatically managed by the RTOS. By adding the overhead induced by the code execution inside the Sampling/Transmitting task, it has been observed that the MCU goes into the Sleeping task as much as 73% of the time, resulting in a measured average power saving of 3.3 mW.

## 3. Experimental Results

In this section, we present the fabricated headstage prototype integrated over a lightweight rigid-Flex PCB, and we present the measured *in vitro* and *in vivo* performances of the whole system.

### 3.1. Rigid-Flex Printed Circuit Board Implementation

**Figure 6:**
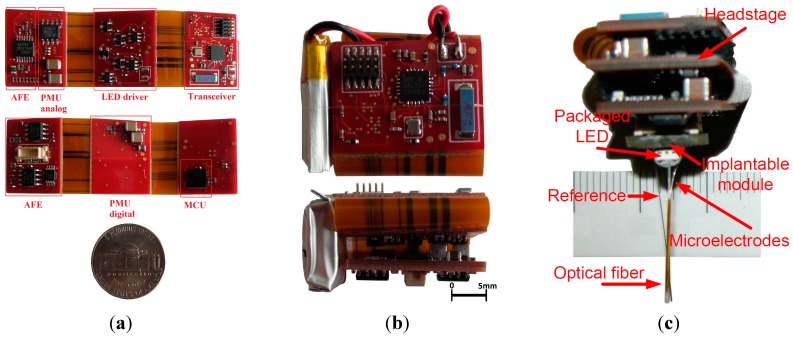
(**a**) Two-sided view of the unfolded rigid-flex PCB; (**b**) The folded system with battery on its side; (**c**) The complete system (folded PCB attached to the implantable module) showing one packaged LED coupled to an implantable optical fiber and two microelectrodes.

Small size and low weight are the most important features of the proposed headstage, so an accurate and precise design for the PCB is crucial. All headstage components are mounted on a rigid-flex PCB containing three rigid sections and two flexible sections which replace any high-volume connectors. The top rigid section is dedicated to the MSP430 and the radio transceiver, the middle rigid section of the PCB contains the PMU and the LED driver components and the bottom rigid section carries the AFE and a Molex SlimStack connector [25] that connects the signal acquisition component to the head-mounted implanted module including the fiber-coupled LEDs and electrodes. The rigid sections are connected to each other using flexible sections where there are no power planes; this results in better isolation between the building blocks and better EMC. In addition, this design eliminates the need for board-to-board connectors which results in lower space occupation. The proposed PCB design is a six-layer PCB with a minimum track width of 0.1 mm and minimum hole size of 0.2 mm. Figure 6 shows the Rigid-Flex PCB from different angles and with the battery and the system mounted on the implantable module, which is a mechanical part that is mounted on the rodent’s head by surgery. Before an experiment with a rodent, the implantable module is first mounted on the rodent head, and then the Rigid-Flex PCB is connected to it via the Molex board-to-board connector. The dimensions of the entire system (excluding the implantable module) when the PCB is completely folded, are 20 mm × 20 mm × 15 mm, and 25 mm × 20 mm × 15 mm with the battery on side. The weight of the headstage without the battery is 4.9 g, the battery itself weights 2.1 g for a total weight of 7 g.

### 3.2. Measured System Characteristics

To test the performance of the AFE, we have uploaded synthetic neural signals made of several action potential shapes taken from a bank into an arbitrary waveform signal generator, and played them at the input of the neural signal recording circuitry of the headstage. The used action potential shapes are taken from [17]. Since the output amplitude of our digital signal generator is much beyond the actual amplitude of the action potentials, a precision resistive divider was used and the overall action potential amplitudes, present at the headstage inputs, varied between less than 50 µV to 150 µV. The outputs of the headstage system are presented later in this section.

Besides the tests involving synthetic action potentials, the frequency response of the AFE was measured with an Agilent 35670A dynamic signal analyzer. Table 1 shows the measured characteristics of the AFE. The frequency response covers from 285 Hz to 6580 Hz. All measurements were made while the LED were activated with typical optical stimulation patterns, and it can be seen that operating the AFE while driving the LED, with high current stimulation signals, does not affect the performance of the AFE. Thus, the high switching currents of the LED do not impose significant noise inside the AFE.

Based on the LED datasheet [20], LED achieve their best performance for a 3.3-V on the LED terminals, which corresponds to a current of 150 mA. Using the high-performance closed-loop current source, a current of exactly 150 mA is passed through the LED, which represents an average current of 15 mA since the maximum duty cycle of the optical patterns is 10%. Table 2 summarizes the characteristics of the optical stimulator.

**Table 1:** Summary of Measured analog front end (AFE) Characteristics.

Parameter	Value
Gain	2851 V/V (69.09 dB)
Low Cut-Off Frequency	285 Hz
High Cut-Off Frequency	6580 Hz
Input-Referred Noise	2.1 µV (rms)
CMRR	110 dB @ 1 KHz
Number of Analog Channels	2
Power Consumption	1 mA @ 3.0 V (3 mW)
Precision	Selectable, 12 or 8 bits

**Table 2:** Optical stimulator characteristics.

Parameter	Value
Forward voltage	3.275 V
Forward current	150 mA
Frequency	1 Hz to 100 Hz
Duty Cycle	10%
Rise Time	1.6 µsec
Fall Time	5.1 µsec
LED input electrical power	491 mW
Implanted fiber output optical power	70 mW/mm^2^

**Figure 7:**
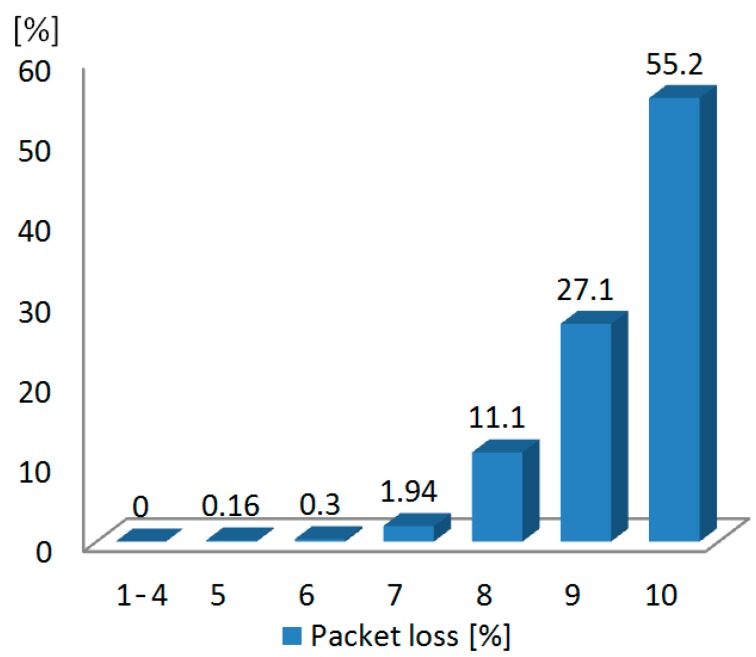
Measured packet losses of the low-power transceiver.

The measured performance of the nRF24L01+ low-power transceiver is summarized in Table 3. Since there is a 250-µs delay between retransmissions of lost or corrupted packets in such a transceiver, automatic packet retransmission was disabled to reach a maximum transmission rate in air of 2 Mbps. Figure 7 shows the amount of packet loss with respect to the distance between the headstage and the base station in an open range field. The tests were carried by progressively moving the headstage away from the base station. In this test, one million packets are sent from the headstage to the base station at 1 m, 2 m, …, 10 m, respectively. Such a test is repeated four times with the headstage antenna oriented in different directions (starting in the direction of the base station with successive rotations of 90°) and the results were averaged. It can be seen that the packet losses are negligible for distances less than 5 m. We observed that the orientation of the antenna affected the results only for distances above 5 m.

**Table 3:** Measured characteristics of the low-power transceiver.

Parameter	Value
Frequency band	2.4 GHz
Modulation	GFSK
In air data rate	2 Mbps
Measured effective data rate	700 Kbps
Transmission power	0 dBm

**Figure 8:**
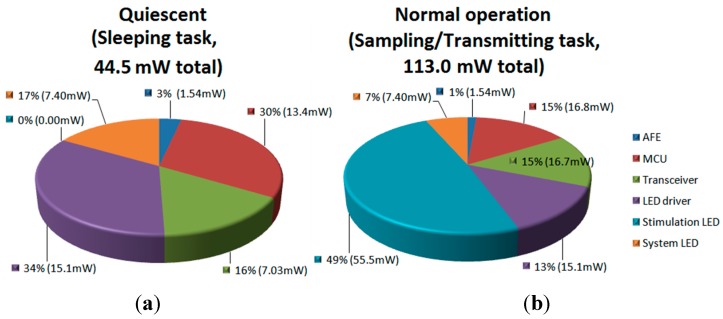
Power consumption of the headstage (V_DD_ = 3.7). (**a**) sleeping task; (**b**) sampling/transmitting task (with optical stimulation enabled).

Figure 8 shows the average power consumption for different building blocks of the system during two tasks, the sleeping task (quiescent) and the sampling/transmitting task (normal operation), with typical stimulation patterns of 100 Hz and 10% duty cycle. The total quiescent power consumption is 44.5 mW, while the total power consumption in normal operation is 113.0 mW. It can be seen that the stimulation LED, on average, consumes almost 50% of the system power. However, when measured instantaneously, the stimulation LEDs consume more than 85% of the battery’s energy. The power consumption of the headstage guaranties 3 h of continuous work with typical stimulation patterns of 100 Hz at 10% duty cycle and 150 mA of LED current, and more than 10 h when the system is executing the Sleeping task, using a 100-mAh nominal current battery, which is more than enough for most experiments. The Lithium-ion battery (Model 051417, MYD Technology CO., LTD, Shenzhen, China) employed in this system has a nominal voltage of 3.7 V, physical dimensions of 5 × 14 × 17 mm^3^, and a weight of 2.1 g. This battery provides a compromise between size, capacity and discharging current.

### 3.3. Test with a Synthetic Input Signal

**Figure 9:**
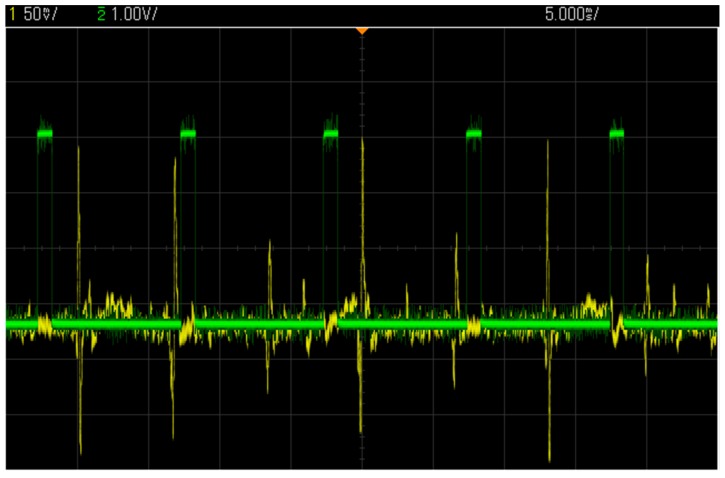
150 µV peak-to-peak spike waveforms at the AFE output along with the stimulation pattern at one LED terminal, both acquired with an oscilloscope.

**Figure 10:**
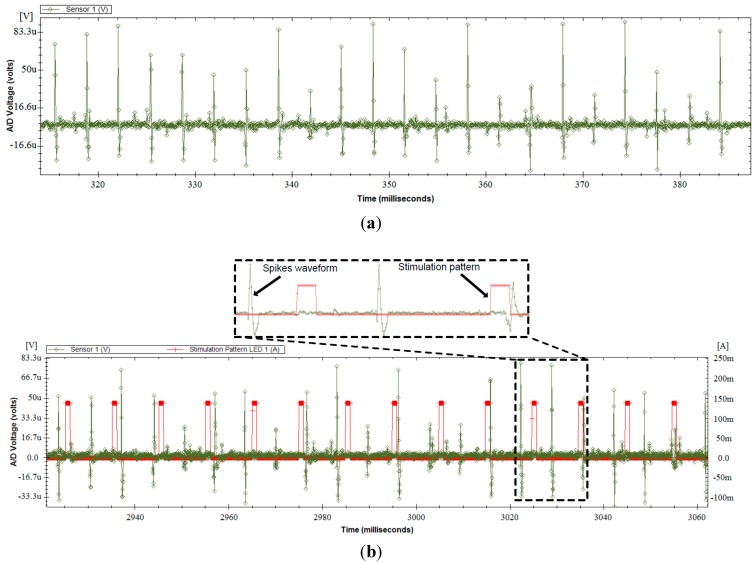
Measurements at the output of the whole data acquisition chain of the headstage fed with a synthetic neural signal input, while optical stimulation is enabled. (**a**) Acquired 150 µV peak-to-peak spike waveforms; (**b**) Output neuronal waveform and stimulation pattern both acquired using the whole data acquisition chain of the headstage.

This section presents the measurement results obtained using synthetic neural signals constructed using real action potential wave shapes, with amplitudes of less than 150 µV, taken from a signal bank. The synthetic neural signals were played at the input of the headstage with an arbitrary function generator. To reproduce realistic experimental conditions, all measurements were performed with optical stimulation enabled with a PWM frequency of 100 Hz and a duty cycle of 10% on two LED channels. Figure 9 shows an oscilloscope view of the AFE output and the voltage at one LED terminal before quantification. It can be observed that the high current of 150 mA, which is flowing into the LED when the PWM is high, induces no visible distortions at the AFE output. Figure 10 presents many neural waveforms acquired on 12 bits using the whole acquisition chain, and recorded in the wireless base station. Figure 10a shows the output of the system for a synthetic input signal including action potentials of around 150 µV of amplitude or less. It can be seen that the inherent noise level of the system is significantly lower than the action potentials. Figure 10b presents an acquired neuronal waveform and the optical stimulation control signal displayed on the same graph. The stimulation pattern that is displayed corresponds to the current flowing into the LED, and was acquired by sampling the voltage across the 0.5-Ω resistor (Figure 3) at the LED driver circuit. Monitoring the stimulation pattern during *in vivo* experiments is critical to see the immediate effect of the optical stimulation on the neural activity, and to make sure that the current flowing into the LED and the generated patterns are the ones expected.

### 3.4. Comparison with Other Wireless COTS Systems

The proposed optogenetic headstage is compared with other recently published COTS components wireless headstages dedicated to neural recording and/or optical stimulation in Table 4. As shown in this table, the proposed headstage is the only system to offer multichannel electrophysiological recording along with multichannel optical stimulation simultaneously. Furthermore, the proposed headstage presents one of the lowest power consumption performance, highest resolution and lowest weight among other reported systems.

**Table 4:** Comparison with other wireless commercial off-the-shelf (COTS) headstages.

Work	No. of Recording Channels	No. of Stimulation Channels	Ch. Sampling Rate (KSample/s)	Bits/Sample (Stim. or Rec.)	Weight (g)	Power Consumption (mW)
This work	2	2 (opt. fiber)	20,000	12	4.9	113
[5]	2	1 (opt. fiber)	20,000	8	7.4	115-475
[26]	1	1	11,700	N/A	8.4	40-120
[27]	1	1	10,000	N/A	N/A	N/A
[28]	1	1	12,000	12	20	N/A
[29]	1	0	N/A	N/A	0.5	N/A
[30]	0	4 (surface LED)	N/A	N/A	N/A	N/A
[8]	0	N/A (µ-ILEDs)	N/A	N/A	N/A	N/A
[31]	15	0	20,000	12	4.0	142
[32]	16	0	25,000	10	16.5	N/A
[8]	16	0	N/A	N/A	3.0	2000
[33]	32	0	30,000	12	N/A	142

### 3.5. In-Vitro Validation

**Figure 11:**
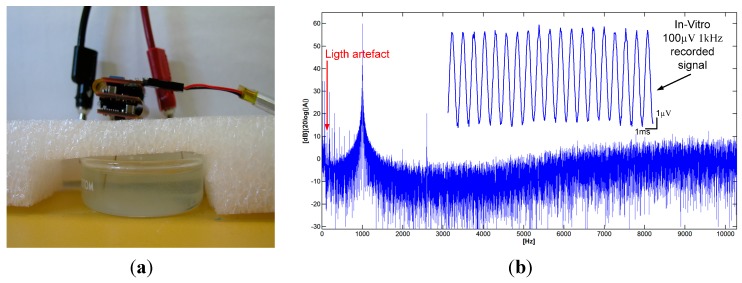
(**a**) *In-vitro* measurement setup with a 1 M saline solution; (**b**) A test waveform recorded on Channel 1 with a microelectrode within the saline solution.

This section presents *in vitro* measurements obtained by plunging the microelectrodes in a saline solution excited with a small AC current. The implantable module described in Section 2.1, including one 200-µm fiber NA = 0.53-coupled to a 470-nm LED along with two microelectrodes passed through a thin polyimide tubing of 400 µm outer diameter, was used in these tests. One microelectrode was dedicated to spike recording, while the other microelectrode was dedicated to local field potential (LFP) recording. Both microelectrodes had a shank diameter of 75 µm and an impedance of 1 MΩ. A third microelectrode, having a shank diameter of 75 µm and an impedance of 0.1 MΩ, was used as a reference electrode. Finally, a fourth electrode with a shank diameter of 100 µm and an impedance of 1 Ω was used for grounding the saline solution (connected to V_body_ in Figure 1). To record LFPs, the bandwidth and the gain of the second AFE channel of the headstage were adjusted to pass and amplify the input signal from 1 Hz to 300 Hz at a gain of 100 V/V. The *in vitro* test setup is presented in Figure 11a, and consists of the headstage system connected with the implantable module having the microelectrodes plunged within a 1 M saline solution excited by a function generator.

Figure 11b shows a recorded waveform from a 100 µV peak-to-peak 1 kHz signal generated between the single-cell microelectrode and the reference microelectrode along with its power spectrum. As can be seen, the low-amplitude input signal is well captured by the implantable module and the headstage. The highest undesirable harmonic amplitude is located at 60 Hz, and posses an amplitude that is 25 dB lower than the input sinewave of 100 µV_pp_ located at 1 kHz, which demonstrates the excellent performance of the headstage. Harmonics resulting from exposition of the tungsten microelectrodes to light [34,35] are also visible in the power spectrum since optical stimulation was activated during the recordings.

### 3.6. In-Vivo Validation

**Figure 12:**
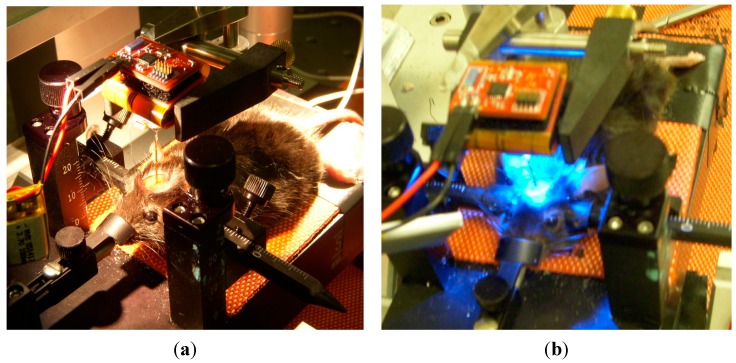
(**a**) Head fixed *in vivo* setup picture; (**b**) The measured *in vivo* data were transmitted between the headstage and a base station located 2 m away.

The performance of the proposed headstage was validated *in vivo* by performing optogenetic stimulations and electrophysiological recordings in the brain of a transgenic mouse expressing Channelrhodopsin-2. Such a proof-of-concept experiment consisted in using the proposed wireless headstage and implantable module to record and stimulate from the brain surface down to a depth of 3 mm of a head fixed mouse (Figure 12). The implantable module was used on the head-fixed animal. The module was not cemented to the skull but was held in place with the headstage by an external attachment tool in order to facilitate the validation procedure.

The animal preparation involved an adult mouse (30–50 g) anesthetized with ketamine/xylazine (10:10 mg/kg) and placed into a mouse stereotaxic apparatus. The mouse line used (Thy1::ChR2-YFP line 4 and Thy1::eNpHR-YFP line 4) [36] was purchased from The Jackson Laboratory. Throughout the experiment, the mouse was maintained under deep anesthesia and its body temperature was maintained at 37.5 °C. To allow probe access to brain tissue, a craniotomy was performed and the dura was removed. The probe was lowered in the cerebral cortex using a micromanipulator. The implantable module including microelectrodes and one implantable LED-coupled fiber described in Section 2.1 was used in this test. All protocols were performed in accordance with guidelines from the Canadian Council on Animal Care.

The optical stimulation patterns were constructed offline using a custom user interface. The optical stimulation pulse width was set between 5 and 20 ms [37]. Stimulation/recording sequences were initiated by transmitting the LED stimulation patterns from the base station to the headstage for producing local brain illuminations via an optical fiber coupled to the LED along with signal acquisition from the microelectrodes. Extracellular electrophysiological signals captured by the microelectrodes were fed to the headstage, and sent to the base station through the wireless data link. The electrode tips (75 µm, 1 MΩ; MicroProbes) were glued 430 µm away from the optical fiber end (200-µm core, NA = 0.53; Doric lenses). The measured light intensity at the tips was 70 mW/mm^2^, which is superior to activation threshold for ChR2 for this type of transgenic mice [37,38].

**Figure 13:**
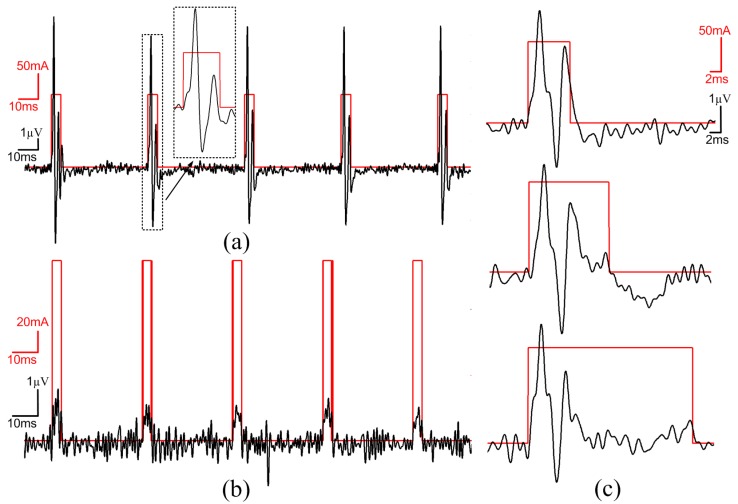
(**a**) Experimental results showing brain extracellular recording (black) in response to local optogenetic light activation (LED current in red), Pulse activations and recorded signals were driven trough the headstage; (**b**) *In vivo* light activation example without action potentials (spikes); (**c**) Example of spiking responses to three different light exposure times (5, 10, 20 ms).

**Figure 14:**
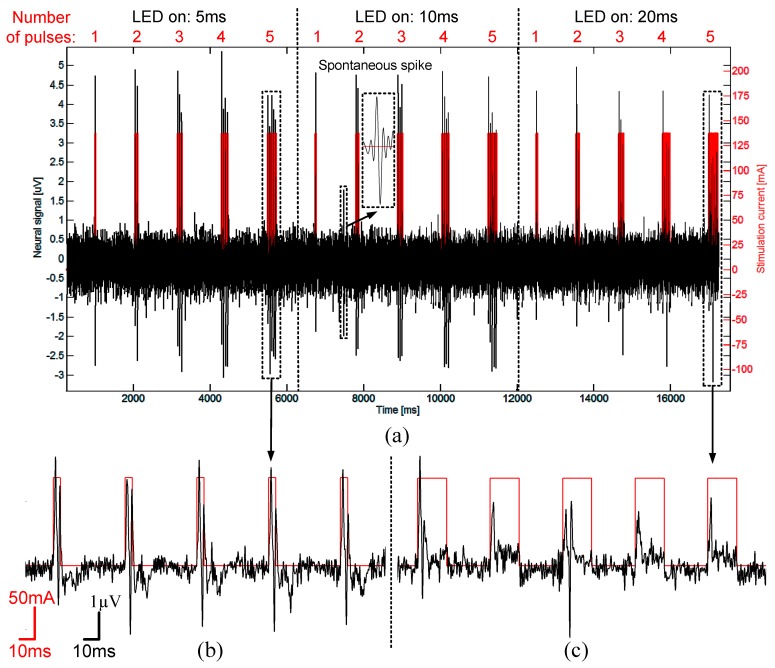
(**a**) Experimental results showing extracellular spike responses to three different pulse sequences (one to five pulses) with different durations per pulse (5, 10, 20 ms); (**b**) Enlargement of a five pulse train (5 ms duration per pulse); (**c**) Enlargement of a five pulse train (20 ms duration per pulse). The longer pulse stimulation paradigm induced progressively failing spiking responses.

As shown in Figure 13 and Figure 14, the proposed system is able to capture and transmit single spike events evoked by light activation and/or from spontaneous firing. The obtained signal-to-noise ratio (SNR) in this *in vivo* configuration ranged from 5 dB to 25 dB for spike amplitudes ranging from 5 to 25 µV, and a sampling rate of 20 kHz enabled clear single unit identifications. Figure 13a shows single spikes evoked by optical stimulation (delay < 1 ms) and was systematically occurring during the stimulation. To show that the recordings were not only artifacts induced by the electrode-light interaction [34,35], we used different illumination patterns (pulses width between 5 ms to 20 ms) to show that the signal did not vary with illumination time (Figure 13c). Spikes with the same shape, were triggered at the same delay and post-spike signals were independent of the light stimulation. We also show in Figure 13b that no evoked spike occurred in regions where there was Channelrhodopsin expression. However, small signal deflections (1.5 µV) were correlated with light pulses illumination. These signals were interpreted as light induced artefacts previously described in literature [34,35].

Figure 14 shows an example of applications where responses to different stimulation paradigms were tested. A train of five pulses of 5 ms duration/pulse induced robust, single spike with each stimulus (Figure 14b). In comparison, a train of five pulses of 20 ms duration induced progressively failing spikes suggestive of building up depolarization block [37] (Figure 14c). The obtained *in vivo* results demonstrate that the proposed wireless system can successfully perform optogenetics stimulation, recording single unit spike and LFP activity.

## 4. Conclusions

In this work, we presented a miniature, wireless optogenetic headstage, which is suitable for recording neural signals from the brains of small animals using two electrophysiological channels. The proposed headstage also has the capability of stimulating the neurons using light pulse trains generated by two implantable fiber-coupled LED. Furthermore, we discussed some of the most probable design issues that designers might encounter while designing such a headstage. These issues are mostly related to electromagnetic compatibility of the headstage where the high-current optical pattern signals can induce noise in the low-amplitude neural signals. Other design characteristics that are also investigated are the small size, low power consumption and quality of signal. The proposed headstage has been validated *in vitro* and *in vivo* in a transgenic mouse expressing Channelrhodopsin-2 in a subset of neurons and proved its ability to trigger neuronal activity with light while recording tens of microvolt amplitude electrophysiological signal with sufficient SNR for accurate action potential (extracellular spike) detection. To the best of our knowledge, the proposed system is the first reported fully wireless headstage to offer simultaneous multichannel optical stimulation along with multichannel brain signal recording capability, which opens up a wide spectrum of research opportunities. Future developments will include further miniaturization and application/testing in freely moving animals. Moreover, on the system design level, we will focus on implementing more neural signal recording channels and more optical stimulation channels. Future challenges also include VLSI integration of all headstage building blocks along with low-power digital signal processing circuitry, as per [39,40], towards more versatile implantable brain-machine-interfacing devices.
